# Internet Gaming Disorder in Adolescents With Psychiatric Disorder: Two Case Reports Using a Developmental Framework

**DOI:** 10.3389/fpsyt.2019.00336

**Published:** 2019-05-10

**Authors:** Xavier Benarous, Pierre Morales, Hanna Mayer, Cosmin Iancu, Yves Edel, David Cohen

**Affiliations:** ^1^Department of Child and Adolescent Psychiatry, Pitié-Salpêtrière Hospital, Paris, France; ^2^INSERM Unit U1105 Research Group for Analysis of the Multimodal Cerebral Function, University of Picardy Jules Verne (UPJV), Amiens, France; ^3^Department of Addiction, Hôpital Pitié-Salpêtrière, Paris, France; ^4^CNRS UMR 7222, Institute for Intelligent Systems and Robotics, Sorbonnes Université, Paris, France

**Keywords:** Internet gaming disorder, gaming misuse, internalizing disorders, externalizing disorders, behavioral addiction, emotional dysregulation, insecure attachment, adolescents

## Abstract

Internet gaming disorder (IGD) has been a controversial entity with various opinions about its clinical relevance as an independent mental disorder. This debate has also included discussions about the relationships between problematic gaming, various psychiatric disorders, and personality traits and dimensions. This paper outlines a developmental-theory based model of Internet gaming misuse inspired by the treatment of two adolescent inpatients. The two clinical vignettes illustrate distinct developmental pathways: an “internalized pathway” *via* the development of social anxiety, emotional and behavioral avoidance; and an “externalized pathway” with a low level of emotional regulation strategies and impulsivity. In both clinical cases, attachment issues played a key role to understand the specific associations of risk and maintaining factors for IGD, and gaming behaviors may be seen as specific forms of maladaptive self-regulatory strategies for these two youths. These clinical observations support the assumption that gaming use problematic in adolescents should be viewed with a developmental approach, including key aspects of emotional development that represent significant targets for therapeutic interventions.

## Background

### Internet Gaming Disorder

In 2013 the American Psychiatric Association included the *Internet gaming disorder* (IGD) in the research appendix of the *Diagnostic and Statistical Manual*, Fifth Edition (DSM-5) recommending that further studies be conducted (1). Following DSM-5 suggestions, gaming disorder (GD) was recently included as a formal diagnostic entity in the 11th edition of the International Classification of Diseases (2) referring to both offline and online games and drawing a distinction between GD and hazardous gaming. The prevalence of IGD/GD is estimated between 1.2% and 5.5% in teenagers, and a problematic gaming use would concern about 1 out of 10 adolescents playing video games (3).

Many concerns have been raised about the identification of the DSM-5 IGD or the CIM-11 GD as discrete clinical entities (4–6). Authors have identified several problems focusing on the diagnostic criteria and their conceptual and empirical issues. These includes the validity of the current diagnostic criteria, the broadening of the disorder to include Internet non-gaming activities (e.g., social media), and the risk of overpathologizing a common activity (3, 6, 7). All that aside, empirical studies have shown that persistent or recurrent gaming behavior is associated with a broad spectrum of psychopathology in adolescents such as social anxiety, depressive disorder, attention deficit disorder, conduct disorder, substance-related addictive disorder, and pathological personality traits (3, 5). These findings are consistent among researches conducted in community-based samples (8–12), Internet-recruited youths (13), and help-seeking populations (14, 15).

Longitudinal studies have supported a bidirectional relation between IGD and mental health problems in adolescents (16–18), e.g., psychopathological traits, such as impulsivity, increase the risk for IGD; in turn, the time of gaming exposure predicts the severity of depressive symptoms 2 years later in adolescents (16).

### A Developmental-Based Model of Internet Gaming Misuse in Adolescents

Adolescence represents a period of vulnerability for the emergence of addictive behaviors with a peak of the incidence during the transition into young adulthood (19). Developmentally, teens are focused on establishing autonomy and identity through sets of social experiences within peer groups. The need to integrate multiple, and somewhat conflicting, demands and developmental needs may result in interpersonal conflicts and emotional distress (20). In this context, addictive behaviors can emerge as a means of developing a new sense of identity within a peer group and relieve emotional distress (21). While the starting point of addictive behavior is often during adolescence, etiological factors are rooted in childhood, especially early-environmental factors and cognitive and socio-emotional dysfunctions (19, 22, 23).

Such as operationalized in the DSM-5, the definition of IGD eludes any developmental perspectives. How do the clinical significance, the natural course, and the therapeutic strategies for IGD vary across age? Indeed, one may think that the impact of severe gaming misuse will depend on how this behavior interferes with normal developmental changes observed at the biological (e.g., cerebral maturation), cognitive (e.g., emotion regulation, motor inhibition), psychological (e.g., identity formation and social roles construction), and environmental (e.g., academic/professional success, peer and family relationship) levels in a specific time window. The developmental view focuses more specifically on *when* and *how* such that vulnerability factors interfere and may form distinct susceptibility pathways to gaming misuse and/or psychopathology.

### Youths With Severe Psychiatric Disorders

Most of the literature devoted to severe gaming misuse in adolescents comes from studies conducted in general populations, Internet-recruited samples, or outpatient clinics. Only anecdotal reports exist concerning youths with severe psychiatric disorders (14, 24). However, in this last group, the aggregation of academic problems, social withdrawal, and the severity of internalized symptoms puts them at very high risk of developing gaming misuse. Moreover, if Internet gaming misuse alters the course of psychiatric symptoms in youths with severe psychiatric disorders, recognizing and treating dual diagnoses would represent a clinically relevant proposal.

### Aims

In this paper, we aimed to describe two case reports of IGD in adolescents with severe psychiatric disorder using a developmental approach. We sought to present different interplays between gaming behavior, psychopathology, and environment. The developmental pathways underlying the association of risk and maintaining factors are discussed for each vignette with regards to existing literature about Internet gaming misuse in adolescents.

## Methods

This study is part of a larger research on the relationship between addictive disorders and psychopathology among adolescents with severe psychiatric disorder (25). Participants are adolescents (12–18 years old) hospitalized in the Department of Child and Adolescent Psychiatry at the Pitié-Salpêtrière University Hospital in Paris. Vignettes have been selected by the psychiatric team and the hospital’s liaison addiction unit. In the remainder of this article, we have used the DSM-5 classification to refer to problematic GD and psychiatric disorders. Written informed consent was obtained from the parents/legal guardians for the publication of these cases. Presentation of the case reports follows the CARE Guideline (26).

## Case Presentation 1

### Patient Information and Clinical Findings

A was a 13-year-old boy referred to the inpatient unit for severe social withdrawal with school dropout since a year and a half. He had no prior psychiatric or medical history. He lived with his identical twin sister and his mother. The father had died 2 years ago from lung cancer. The twins were born prematurely at 34 weeks, but no delay in psychomotor acquisitions was reported.

Following the death of his father, A started to develop isolation and social withdrawal. Around the same period, he started playing at a construction game on his computer. The time spent in this activity increased, and the patient gave up school and other activities. Over the past year, A played 10 to 12 h per day with no period free of playing longer than 1 day. When not gaming, A was irritable, vindictive, and verbally aggressive. In addition, gaming did not involve any socializing aspects (e.g., forum or online competition). During the last 6 months, he was completely confined to his room (except for personal hygiene) spending almost all of the daytime playing the video game. All the family’s attempts to help him reduce gaming failed. The patient actively refused to meet mental health professionals, and during home visits, he stayed locked in his room.

### Diagnostic and Psychopathological Assessment

At admission, the patient appeared to be a discrete boy. He looked sad and withdrawn with minimal verbal interaction. The speech was monotone and overly soft with many pauses and, in particular, reluctant to talk about his thoughts. A was particularly careful to pick the right word to answer questions. He expressed a pervasive feeling of hollowness and a loss of interest in his surroundings. His mood was poorly influenced by external circumstances. He described the feeling as being emotionally paralyzed rather than sadness. A reported no pessimistic thoughts or feelings of hopelessness; however, he was unable to project himself into the future and had no motivation to perform any activities other than gaming. Sleep and appetite were preserved and no delusion was reported. The diagnosis of persistent depressive disorder (F34.1) was made (1).

Prior to the onset of the current depressive disorder, A experienced socio-emotional and interpersonal difficulties. He shared his emotional experiences only on rare occasions and was reluctant to seek support for basic or emotional needs. As a child he is described as frequently embarrassed in new and unfamiliar situations, with few behavioral strategies to manage his emotion. The restriction of facial and voice affect, initially interpreted as a sign of depressive mood, was reported since an early age.

During medical interviews, A’s mother presented poor emotional insight. Her voice and face expressed deep sadness, but she was reluctant to discuss her feelings. Questions about the relations between family grief, the impact on each family member, and A’s psychiatric symptoms were eluded. She never mentioned her own social phobia that we discovered long after this hospitalization. In fact, it turned out that the weekly appointments to the adolescent outpatient care service were her only source of relational contacts. About gaming, she felt helpless in monitoring the gaming use. She agreed to receive behavioral guidance but never managed to apply any suggestions. Her motivation to change the current situation at home seemed low.

### Therapeutic Interventions, Follow-Up, and Outcomes

A was treated with an antidepressant, a selective serotonin reuptake inhibitor (SSRI), sertraline up to 75 mg/day. In the ward, he was involved in different activities with other inpatients in view of promoting positive experiences with adults and peers. He seemed more open and talkative with the paramedical staff and with other youths than during the medical interviews. He had a weekly support group and a group for behavioral and substance-related addictive disorders. The patient started school readaptation a few hours per day.

After 4 weeks, the patient felt progressively better. During permissions at home, A is described as more dynamic and emotionally reactive. He started to enjoy usual interests with other members of the family and actively sought out friendship in planning lunch during the weekend with adolescents met at the hospital. Progressively, he spent less time playing video games (around 2 h per day) without anxiety when not playing.

Despite the clinical and functional improvement, both A and his mother seemed unable to identify external or internal factors that contributed to A’s depressive disorder and gaming misuse. They did not express any worries regarding possible relapse. For both of them, mental projections into the past or the future were nearly impossible or were unrealistic. For example, despite a year and a half without being at school, A and his mother declined all school adaptations. The patient viewed grade repetition as a source of stigmatization and refused to return to school. Further, therapeutic suggestions such as daily care intervention or individual psychotherapy were politely declined by the patient.

After discharge, the patient had regular appointments in an outpatient care structure and started in a new school. After 10 weeks, the mother contacted us to explain that her son refused to follow outpatient care, no longer attended school, and again had social withdrawal with severe gaming misuse.

## Clinical Relevance

### Interplay Between Emotional Distress and Gaming Misuse

In this vignette, anxiety/mood symptoms and Internet gaming misuse are highly correlated: a decrease in the severity of mood symptoms was associated with less gaming behavior, and the “relapse” into severe gaming occurred with the resurgence of emotional distress. Such association has been well demonstrated (8, 10, 11). In longitudinal studies, pathological video game use is predicted by anxiety (including social phobia) and depressive symptoms (9, 16, 17). Such bidirectional interplay between gaming misuse and anxiety/mood symptoms can progressively generate an ongoing cycle of internalizing symptoms (18).

### Insecure Attachment as a Shared Vulnerability Factor

Here, we made a diagnosis of associated reactive attachment disorder (F94.1) (1) with regards to A’s difficulties to initiate and respond to most social interactions in a developmentally normal way continuously observed since his early childhood. Moreover, a context of caregiving emotional deprivation was very likely considering the difficulties for the mother to recognize and make sense of her own emotions and those of her children.

Among children with insecure attachment style, an anxious-avoidant subtype has been identified (27). These children tend to not exhibit distress on separation and either ignore the caregiver or turned away from him/her on their return. Main (28) suggested that these children actively avoided a consistently unresponsive caregiver in view of avoiding a situation of distress and ultimately maintaining a sense of control. The avoidance of any new relational situation in children with an anxious-avoidant attachment type can lead to poor self-esteem and internalizing symptoms *via* the lack of opportunities to learn social skills with his/her caregiver (29).

Adolescents and young adults with problematic Internet use are more likely to have an insecure attachment style (30–33). An Italian study found that attachment styles contribute for a significant proportion (13%) to the variance in scores of addictive behaviors in college students (33). Some psychological traits reported in this clinical vignette, such as the high-level of psycho-rigidity, mental and interpersonal control, and relational inflexibility, are also reported as a putative risk factor for the onset and maintenance of gaming misuse in adolescents (3, 33). One study supports this developmental view, as authors found that attachment/personality traits in young adults mediate the impact of dysfunctional family relationships on the occurrence of IGD (34). In the Discussion, we detail how avoidance and social withdrawal as a persistent maladaptive reaction in a patient with an anxious-avoidant insecure attachment play a key role in the emergence and persistence of the mood disorder and gaming problematic.

## Case Presentation 2

### Patient Information and Clinical Findings

B was a 15-year-old boy referred to an inpatient unit for severe disruptive behaviors after being expelled from his school. He lived with his 10-year-old younger brother and two half-brothers (aged 20 and 30 years). The parents were separated although living together. B had commonly been exposed to severe arguing and fighting between them. Both parents were unemployed. The father had an untreated alcohol addiction and the mother had no specific past psychiatric history. The family had been followed by social services since B was 3.

The patient’s pregnancy was complicated by gestational diabetes and occasional maternal alcohol intake. B was born prematurely at 35 weeks of gestation. He had a delayed onset of speech (first words at 2 years) and fine motor difficulties. At entrance in first grade, he had difficulties understanding verbal instructions and performing graphomotor activities. Distractibility and emotional dysregulation were also noted. At age 6, a Wechsler Preschool and Primary Scale of Intelligence (WPPSI-III) test found a heterogeneous functioning in normal range (Verbal IQ = 100, Performance IQ = 75). At age 7, the patient was addressed to a foster care family with a full-time inclusion in an educational facility for youths with behavioral problems. Improvement in emotional control was noted.

At age 13, B faced multiple adverse life events (incarceration of his half-brother, left foster care to return to family home, and change in the pedagogic team). He became physically aggressive against peers and adults with several rage outbreaks per day. Different medications were tried with no or partial improvement: tiapridum (a first generation antipsychotic) up to 15 mg/day, carbamazepine up to 200 mg/day, risperidone gradually increased to 4 mg/day. B was excluded from his educational facility following the aggression of a member of the educational staff. Since then, the patient has stayed at home all day. He was described as severely irritable with multi-daily outbursts of uncontrollable anger. He was verbally and physically aggressive against his parents in a context of frustration and tried to strangle a neighbor after a banal remark. During this period, B maintained his interests in his usual activities, for example, caring for animals or cooking.

He drastically increased time on his computer following school expulsion. He mostly played Role-Playing Games and First Person Shooter Games, with violent scenarios. Daily playing sessions lasted 2–6 h, occasionally during the night. He could compulsively watch online videos during several hours, either childish cartoons or violent videos of aggression. B had daily alcohol consumption usually alone of one glass of wine or a can of beer with binge-drinking sessions almost every month (i.e., 10 g of alcohol each day or 8.75 units per week on average). He explained that alcohol was a means to “calm down.” Of note, the patient was very critical of his father’s addiction problem, criticizing his father’s inability when drunk to care for him. He also had very occasional cannabis use (smoked one joint every 2 months).

### Diagnostic and Psychopathological Assessment

During individual interviews, B was calm. He described a feeling of hostility, persisting anger and ambivalent feelings toward adults (“worry, shame and anger at the same time”). He reported being exposed to violent conflicts at home and frequently having to take care of his drunk father. Globally, he described a situation of physical and emotional neglect at home. B expressed concern about the consequences of his behavior and his future (he wished to become a cook). He was afraid of “being always angry” after leaving the hospital or that similar problems would repeat with his young brother. Sleep and appetite were preserved.

In the unit, he had few contacts with other youths. He was too clumsy to be involved in sport activities and was often rejected by the group when playing board games. He felt more comfortable with younger patients with whom he shared common interest in animals. When he felt worried, the patient sought attention from adults with provocative behaviors or threats. He could suddenly give a blow to a wall, against a window, or against a piece of furniture without any explanation.

Psychomotor assessment showed evidence of a developmental coordination disorder (F82) (1): general motor and coordination test score was at 0.1 percentile, visuomotor integration test score was very low, and he had −7 standard deviations for writing abilities (**Table 1**). Language evaluation showed evidence of severe dyslexia (Reading disorder, F315.0) with normal to weak abilities in oral language but very deficient reading competency (**Table 2**). The diagnosis of disruptive mood dysregulation disorder (F34.8) in an adolescent with multiple learning disabilities (developmental coordination disorder, dyslexia, dysgraphia) was set up and explained to the patient and his parents.

**Table 1:** Psychomotor assessment performed by B.

Tasks	Scores
**Gross Motor Skills: M-ABC-2**	
Manual dexterity sub-score	14 (1^st^ %ile)
Ball skills sub-score	14 (16^th^ %ile)
Static and dynamic balance sub-score	9 (0.1^st^ %ile)
Total score	37 (0.1^st^ %ile)
**Gnosopraxis: EMG**	
Hands movements imitation	7.5/10 (−2.98 SD)
Fingers movements imitation	3/16 (+0.42 SD)
**Bodily Image**	
GHDT test	DA = 7.25 years
Berges somatognosia test	Succeed
**Visual perception and visual-motor integration skill: DTPV-2**	
Motor-reduced visual perception	36 (32^nd^ %ile)
Visual-motor integration	27 (27^th^ %ile)
**Graphism**	
BHK-ado	37 (−7 SD)
Bender visual-motor test	DA = 6.0 years
**Rhythm tasks**	
Auditory-perceptual-motor task (Soubiran)	Failed
Auditory-visual-kinesthetic task (Soubiran)	Failed
Tapping (Stambak)	Failed

DA, developmental age; SD, standard deviation; M-ABC, Movement Assessment Battery for Children; EMG, Evaluation de la Motricité Gnosopraxique; GHDT, Goodenough–Harris Drawing Test; DTPV-2, Developmental Test of Visual Perception 2^nd^ edition; BHK-ado, Bender test, Bender Visual-Motor Gestalt Test.

**Table 2:** Cognitive, oral, and written language assessments performed by B.

Tasks	Scores
**Wechsler intelligence scale for children-IV**	
Verbal comprehension index	
Perceptual reasoning index	
Working memory index	
Processing speed index	
**Phonology**	
Repetition monosyllabic (EDA)	DA = 6 years
Suppression of the last phonem (EDA)	DA = 9 years
**Semantic**	
Lexical reception (EDA)	DA = 9 years
Picture designation (EVIP)	DA = 13 years
Picture denomination (EDA)	DA = 9 years
Semantic fluency (DEN 48)	- 1.9 SD compared to 8^th^ grade sample
**Morphosyntax**	
Syntax understanding (EDA)	DA = 9 years
Sentence completure (EDA)	DA = 9 years
**Reading**	
Reading words in 1 min (LUM)	- 1.6 SD compared to 2^nd^ grade sample
Reading text	DA = 6 years
**Writing**	
Figure copy (L2MA2)	- 1 ET compared to 6^th^ grade sample
Text transcription	DA = 6 years

EDA, Examen des Dyslexies Acquises; EVIP, Échelle de vocabulaire en images Peabody; DEN 48, Epreuve de dénomination pour enfants; LUM, Lecture en Une Minute; L2MA2, spoken language, written language, memory, attention.

### Therapeutic Interventions, Follow-Up, and Outcomes

The treatment with carbamazepine was discontinued and risperidone was decreased to 2 mg/day, a dose more typically used in youths with disruptive behaviors (35). A benzodiazepine, diazepam, was added for its anxiolytic effect. The patient also started a psychomotor rehabilitation in the service (weekly group relaxation and individual sessions). The need for an intensive speech therapy was explained to the parents. Collaboration with social services was of main importance in this hospitalization. He was accompanied to a juvenile court session where a placement decision was set up. During the last week of the hospitalization, he visited a new residential care facility.

A major clinical improvement was observed during the hospitalization with a decrease in the behavior problems. At discharge, B no longer presented diagnostic criteria for IGD, and no specific intervention was required. Six months later, B no longer presented clinical or functional impairment.

## Clinical Relevance

### Interplay Between Disruptive Behaviors and Gaming Misuse

We found in this vignette a relation between disruptive behaviors and gaming misuse in line with preexisting literature in adolescents (4, 8, 10, 11, 15). A Spanish study showed that disruptive behavioral disorder was the most frequent diagnosis associated with IGD in a clinical sample of youths (15). It seems that IGD is associated with both proactive and reactive (impulsive) types of aggressive behaviors in adolescents. Wartberg et al. (10) found that in a large community-based sample of adolescents, those who self-reported symptoms for IGD were more prone to anger control problems, antisocial behavior, and SDQ hyperactivity/inattention subscale, in multivariate analysis.

### Insecure Attachment, Emotional Dysregulation, and Impulsivity

The description of patient B’s usual way of dealing with emotional stressors since his early childhood was strongly evocative of an anxious-resistant subtype of attachment disorder (also called ambivalent attachment). Children with an anxious-resistant subtype of attachment disorder exhibit a high level of distress on separation and tend to be ambivalent when his/her caregiver returns (27). In middle childhood, these children are more likely to adopt “controlling” behavior (i.e., role-reversed) on caregivers. The displays of anger or helplessness towards the caregiver on reunion have been regarded as a strategy for maintaining the availability of the caregiver by preemptively taking control of the interaction (36).

A persistent lack of predictability of the caregiver’s responses, such as found in B’s family, did not allow children to develop reliable expectations about adults’ behaviors. As a consequence, these children did not develop a proper sense of trust in their own ability to interpret their social world: they have, in general, more difficulties to accurately anticipate and interpret emotional cues (e.g., facial expression) and to understand their own mental state (37).

The fact that these children are immersed in a social world unintelligible to them and have more difficulties to stay “attuned” to others’ emotional state explained the difficulties to develop optimal emotional regulation strategies and the myriad of associated behavioral problems (e.g., oppositional behavior, poor tolerance to frustration, temper tantrums, impulsive aggressive behaviors, peer rejection) (29, 36).

A low level of emotional regulation skills in childhood is a significant risk factor for behavioral addictive disorders in adolescents, including GD and Internet-related disorders (11, 22, 23, 32). Youths with difficulties to regulate their emotions could engage in such repeated behaviors to avoid or regulate negative feelings and emotions or to prolong positive emotional states (38). In the Discussion, we explain how poor emotional regulation strategies could represent both shared vulnerability factors and mediators of the relation between psychopathology and gaming misuse in the patient.

## Discussion

### Internalized Pathway to Gaming Misuse

**Figure 1:**
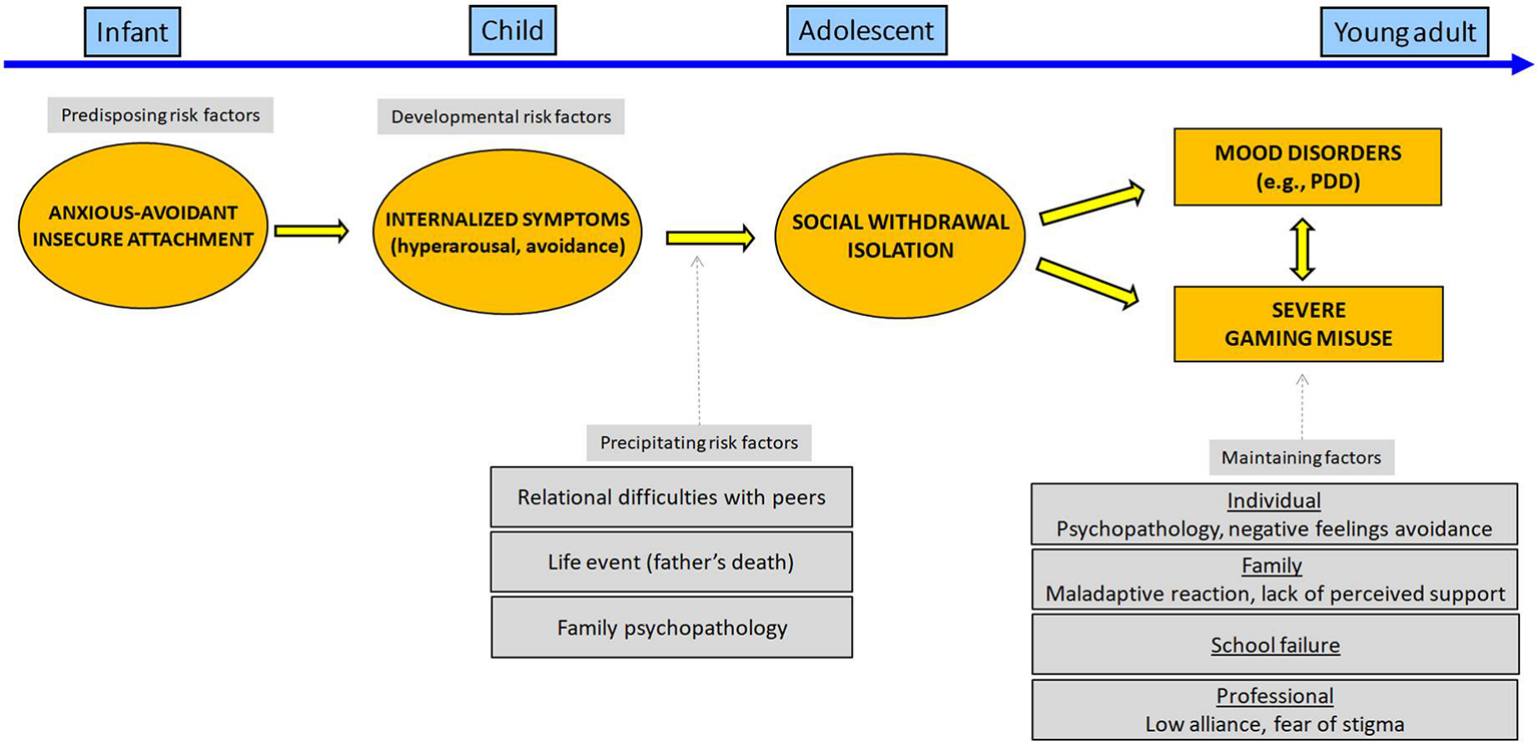
Developmental pathway leading to severe gaming use for patient A.

We present in **Figure 1** a comprehensive view of the relation between risk and maintaining factors for video gaming misuse for patient A. We hypothesized that a) the anxious-avoidant insecure attachment style as an infant, b) the internalized symptoms in childhood, and c) the persistent depressive disorder in early adolescence were distinct behavioral expressions of a common developmental pathway for liability for anxiety/mood disorders. In a context of individual vulnerability and poorly adjusted environment, our patient had through childhood poorly effective coping strategies to manage emotional distress. During adolescence, familial adverse events (loss of paternal support, maternal depression) and the difficulties with peer relationships made it more difficult for him to turn to a peer group to establish a new sense of identity and intimacy.

Gaming can be regarded here as a maladaptive coping strategy to avoid interpersonal relationships seen as frightening or unpredictable, while our patient favors the immediate gratification of gaming as an attachment alternative to relationships. To paraphrase Flores (39), gaming acts “*as both an obstacle to and a substitute for interpersonal relationships*.” In turn, excessive gaming results and its relational consequences both worsen self-esteem and fuel depressive mood. The combination of gaming-related positive expectancies and behavioral/emotional avoidance for the development of IGD seems likely in this context as has been shown among adults (40).

### Externalized Pathway to Gaming Misuse

**Figure 2:**
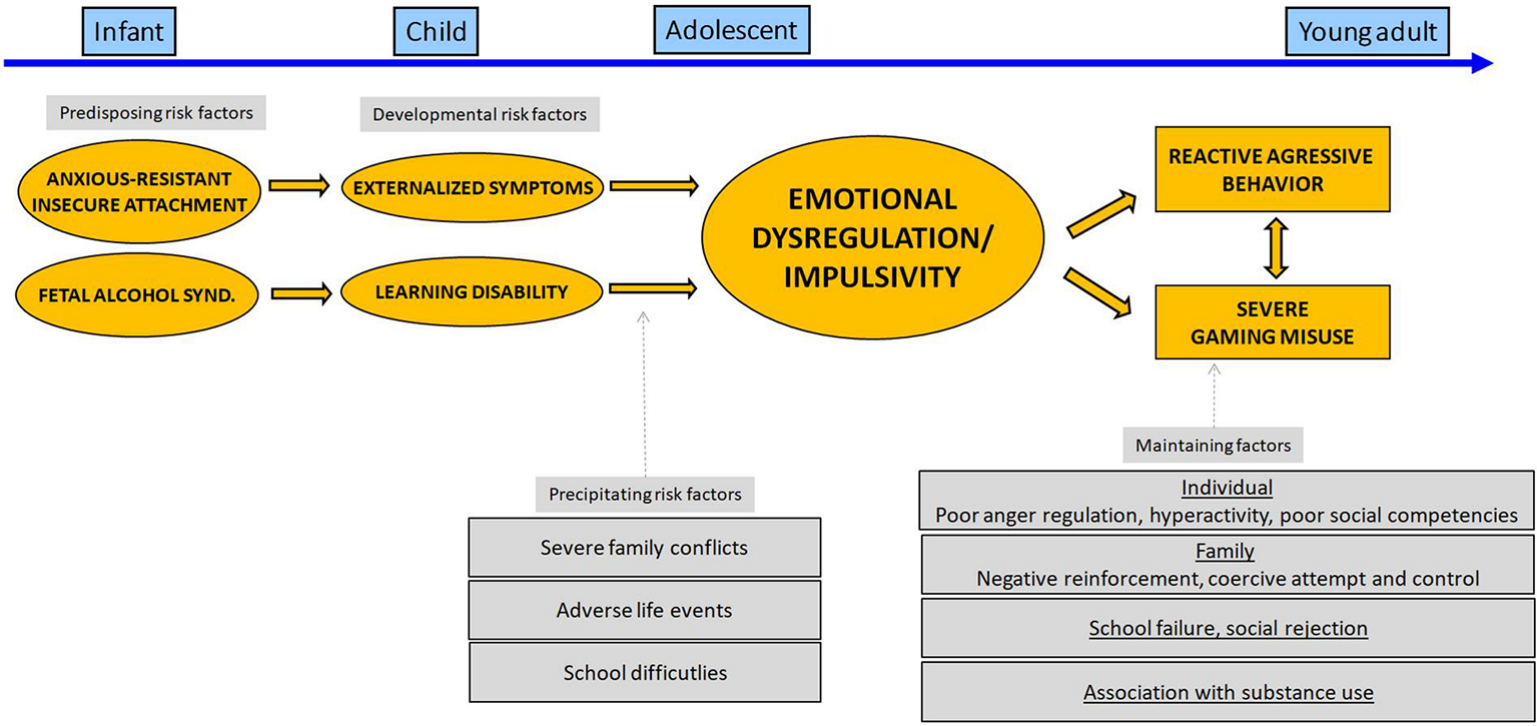
Developmental pathway leading to severe gaming use for patient B.

We present in **Figure 2** a distinct developmental pathway leading to gaming misuse. We hypothesized that a) school difficulties, especially in a context of learning disabilities, and b) environmental adversity, including lack of parental support and parental supervision, were important precipitating risk factors for both externalizing behaviors and gaming misuse. While cognitive difficulties such as delay in executive function development had existed since preschool age, its impact in terms of socio-emotional abilities may worsen with age in a context of increasing social and academic expectations. It is very likely that the difficulties in cognitive and motor inhibition to delay immediate reward generated multiple stressful situations (e.g., in school, in family) that fueled the patient’s feeling of distress, frustration, and resent leading to “developmental cascades” (41). In adult literature such difficulties seem underpinned with abnormal prefrontal activities during the resting state (42) and delay tasks (43).

Early environmental or genetic factors that affect neurological and cognitive maturation may play roles in the emergence of psychopathology and gaming use problematic in this vignette. Firstly, genetic factors could be implied given that B’s father was diagnosed with alcohol use disorder and the overlap between the genetic factors associated with behavioral and substance-related addictions (44). Secondly, fetal alcohol exposure may have interfered with B’s developing central nervous system leading to suboptimal prefrontal cognitive activities and thus to faulty inhibitory control. Thirdly, early traumatic experiences and emotional neglect could also contribute to impede neurological maturation and cognitive abilities (45).

In this case report, we may hypothesize that B’s compulsive search for an object of immediate pleasure through gaming may have resulted from maladaptive self-regulation strategies in a context where other forms of emotional self-regulation strategies (e.g., cognitive appraisal, seeking support) are inefficient. Using a psychodynamic view, gaming behavior can be regarded as a substitute for other common sources of pleasure at this age at an object level (e.g., poor family and peer relationship) and a narcissistic level (low self-gratification in a context of failure/poor academic or educational performance) (46, 47). The limitation of B’s affective domain to gaming may be partly explained by the necessity to restrict possible sources of pleasure/displeasure to limited and thus predictable factors in his environment. The rules of the video game are probably easily understandable for B and viewed as more “fair” than external rules.

### Clinical and Research Implications

A’s difficulties to recognize his own feelings and express conflicting views about care, usual for adolescents with attachment issues, complicate therapeutic relationships and treatment plan adhesion (48). A low level of treatment motivation and readiness to change are regarded as the main reasons for the lack of effectiveness of psychotherapy in adolescents with IGD (49, 50). Insight-oriented psychotherapies may be of main interest for adolescents with IGD such as attachment-based psychotherapy (20), mentalizing-based psychotherapy (51), and dialectical-behavioral therapy (52). Such approaches promote patient emotional awareness and expression (e.g., for A) or gaining a sense of trust in relationships (e.g., for B) that contribute to an increased proneness for multiple co-occurring addictions (53).

What is the role of hospitalization in this context? A’s separation from his usual environment helped him to break out of the accustomed pattern of excessive gaming, but a relapse occurred shortly after hospital discharge. Hospitalization of adolescents with behavioral addiction is not only an opportunity to stop the maladaptive behavior but also to improve the adolescent’s and his/her family’s knowledge about the internal and external maintaining risk factors (54). As shown here, the attachment issue is frequently associated with family factors for IGD that could deserve targeted interventions: parental depression (55), parental anxiety (10), poor level of perceived family support (56), or parental insecure attachment (32, 33).

Some have suggested that family difficulties may have a more causative role in the emergence of IGD in adolescents. Young people with problematic Internet use had greater disapproval of their families and perceived their parents as less supportive and warm when compared with young people with no problematic Internet use (57). Xu et al. (58) found in a sample of 5,122 adolescents that the quality of parent–adolescent relationship and communication was closely associated with the development of adolescent Internet addiction. For Lam (55), Internet misuse could be seen as an attempt to compensate problematic interactions with one parent, especially in case of parental psychopathology. In a context of severe emotional neglect, as in B’s family, video gaming seems to be one of the only stable and predictable sources of pleasure in a family where adults were poorly involved and available for their children.

Finally, as illustrated in these two clinical cases, careful assessment of environmental backgrounds and developmental history is of major importance to find ongoing stressful factors that fuel the patient’s psychopathology and/or maladaptive emotional regulation strategies. Youths with multiple specific learning disabilities may represent a very high risk population for IGD considering the multiple risk factors for gaming misuse, e.g., academic failure, lower socio-emotional competencies, and delay in executive function development.

## Conclusion

We stress the need to consider the developmental pathways underlying the association between psychopathology and/or gaming misuse in youths with IGD. An “internalized” and “externalized” pathway to gaming misuse *via* the onset of distinct, but somewhat overlapping, psychiatric disorders and environmental factors is presented in **Figures 1** and **2**. Gaming behaviors may be seen as specific forms of maladaptive self-regulatory strategies in youths with attachment issues. Considering underlying vulnerability factors, such as insecure attachment style and emotional dysregulation, may represent an important therapeutic opportunity for youths with dual disorders.

## Author Contributions

XB and DC performed substantial contributions to the conception and design of the work. XB, PM, CI, and HM performed substantial contributions to the acquisition, analysis, or interpretation of data. XB drafted the work or revised it critically for important intellectual content. XB, PM, YE, DC, CI, and HM gave the final approval of the version to be published. XB, PM, YE, DC, CI, and HM agreed to be accountable for all aspects of the work in ensuring that questions related to the accuracy or integrity of any part of the work are appropriately investigated and resolved.

## Funding

We sincerely thank the institutions that have financially supported this project: la Direction General de la Santé (DGS), la Caisse Nationale de l’Assurance Maladie des Travailleurs Salariés (CNAMTS), la Mission interministérielle de lutte contre les drogues et les conduites addictives (MILDECA), and l’Observatoire national des Jeux (ODJ) (“IReSP-15-Prevention-11”).

## Conflict of Interest Statement

The research was conducted in the absence of any commercial or financial relationships that could be construed as a potential conflict of interest.
